# Entomological outcomes of cluster-randomised, community-driven dengue vector-suppression interventions in Kampong Cham province, Cambodia

**DOI:** 10.1371/journal.pntd.0010028

**Published:** 2022-01-25

**Authors:** Jacob Bigio, Leo Braack, Thy Chea, Srun Set, Sokha Suon, Pierre Echaubard, John Hustedt, Mark Debackere, Bernadette Ramirez, Didot Budi Prasetyo, Sam Bunleng, Alexandra Wharton-Smith, Jeffrey Hii

**Affiliations:** 1 Research Institute of the McGill University Health Centre, Montreal, Canada; 2 McGill International TB Centre, Montreal, Canada; 3 Malaria Consortium, Faculty of Tropical Medicine, Mahidol University, Bangkok, Thailand; 4 UP Institute for Sustainable Malaria Control, University of Pretoria, Pretoria, South Africa; 5 Malaria Consortium, Phnom Penh, Cambodia; 6 SOAS, University of London, London, United Kingdom; 7 London School of Hygiene and Tropical Medicine, London, United Kingdom; 8 Special Programme for Research and Training in Tropical Diseases (TDR), World Health Organization, Geneva, Switzerland; 9 Consultant Entomologist, AC Investment, Phnom Penh, Cambodia; 10 National Center for Parasitology, Entomology and Malaria Control, Ministry of Health, Phnom Penh, Cambodia; 11 Department for Global Health and Development, Faculty of Public Health and Policy, London School of Hygiene and Tropical Medicine, London, United Kingdom; 12 College of Public Health, Medical and Veterinary Sciences, Division of Tropical Health and Medicine, James Cook University, Australia; Centers for Disease Control and Prevention, Puerto Rico, UNITED STATES

## Abstract

Cambodia has one of the highest dengue infection rates in Southeast Asia. Here we report quantitative entomological results of a large-scale cluster-randomised trial assessing the impact on vector populations of a package of vector control interventions including larvivorous guppy fish in household water containers, mosquito trapping with gravid-ovitraps, solid waste management, breeding-container coverage through community education and engagement for behavioural change, particularly through the participation of school children. These activities resulted in major reductions in Container Index, House Index, Breteau Index, Pupal Index and Adult Index (all p-values 0.002 or lower) in the Intervention Arm compared with the Control Arm in a series of household surveys conducted over a follow-up period of more than one year, although the project was not able to measure the longer-term sustainability of the interventions. Despite comparative reductions in Adult Index between the study arms, the Adult Index was higher in the Intervention Arm in the final household survey than in the first household survey. This package of biophysical and community engagement interventions was highly effective in reducing entomological indices for dengue compared with the control group, but caution is required in extrapolating the reduction in household Adult Index to a reduction in the overall population of adult Aedes mosquitoes, and in interpreting the relationship between a reduction in entomological indices and a reduction in the number of dengue cases. The package of interventions should be trialled in other locations.

## Author summary

Dengue is a virus transmitted by mosquitoes which causes unpleasant flu-like symptoms and has no specific treatment. In Cambodia, mosquitoes which can carry dengue virus breed in fresh water collected in containers such as the large clay jars used for domestic water storage in the many rural parts of the country which lack piped household water supply. This study examined a package of interventions including putting mosquito larvae-eating guppy fish in household water containers, trapping adult mosquitoes, removing mosquito breeding sites from around houses and providing community education about dengue, particularly to school children. The study found that implementing this package of interventions significantly reduced the number of household water containers which contained mosquito immature stages, the average number of mosquito pupae per house and the average number of adult mosquitoes per house when compared with a control group which did not implement the package. Though these results are promising, it is not certain that these reductions will directly translate to a reduction in the number of people in the study areas becoming infected with dengue and more studies are needed to investigate this link.

## Introduction

Dengue is the most common and widely distributed human arbovirus, with an estimated 390 million infections and 90 million cases per year [1]. There is no specific treatment for dengue and the use of a vaccine developed by Sanofi Pasteur was widely discontinued due to safety concerns [2–4], though it is approved for use by the European Medicines Agency in people aged 9 to 45 years [5] and by the United States Food and Drug Administration in children aged 9 to 16 years, with both regulators allowing its use only in individuals with confirmed previous dengue infection living in endemic regions [6]. Vector control methods remain the mainstay of dengue control.

Cambodia has one of the highest dengue infection rates in Southeast Asia, with an average 103 cases per 10,000 population reported annually to the national surveillance system [7]. Dengue in Cambodia is transmitted by both *Aedes aegypti* and *Aedes albopictus*. The mosquitoes breed in fresh water collected in a variety of containers, particularly the large clay or concrete jars, basins and tanks used for domestic water storage in the many parts of rural Cambodia which lack piped household water supply, but commonly also in discarded tins, bottles, coconut husks, old tyres or any container capable of holding even a small quantity of rain-water [8,9]. Vector control strategies in Cambodia have focused on the use of the larvicide temephos in these household water containers and on thermal fogging with pyrethroids [10,11]. However, work showing widespread resistance to deltamethrin, permethrin and temephos among *Aedes aegypti* in Cambodia suggests a need for control strategies which do not rely upon these insecticides [12].

A trial of *Mesocyclops*, a genus of larvae-eating copepods that was used in a successful local elimination campaign for *Aedes aegypti* in several regions of Vietnam, proved ineffective in household water containers in Cambodia, possibly for climatic and ecological reasons [9,13]. *Bacillus thuringiensis israelensis* (*Bti*), a facultative anaerobic Gram-positive bacterium which produces toxins that kill immature mosquito stages and is available as a water-dispersible granule, showed promise in two trials in Cambodia [14,15] and is being evaluated further by the Cambodian government [9]. The larvicide pyriproxyfen, sold under the trade name Sumilarv, showed promise in a 2008 field trial and proved effective in a recent cluster-randomised trial in Cambodia [16,17]. A further biological control strategy is the use of larvivorous guppy fish in water containers. Guppy fish are highly effective predators of mosquito larvae [18,19] and early studies of the species *Poecilia reticulata* in Cambodia showed promising results for community-based dengue vector control [20,21].

A cluster-randomised trial of the use of guppy fish was conducted in Kampong Cham province in Cambodia over one year from 2015–16, with results published by Hustedt et al. (2021) [17]. It was a three-arm trial comparing entomological outcomes in a control arm, an intervention arm which used guppy fish in combination with Communication for Behavioural Impact (COMBI) activities, and an intervention arm which additionally used Sumilarv in combination with guppy fish and COMBI activities. Both intervention arms showed statistically significant reductions in the number of adult female *Aedes* mosquitoes trapped per house, as well as significant reductions in indices of immature mosquito stages, when compared with the control arm.

Here we report the entomological outcomes of a separate cluster-randomised trial of a much more substantial package of interventions, incorporating biophysical and community engagement interventions, which was conducted in many of the the same villages in Kampong Cham province over two years from 2018–20.

### Objective

The objective of this second trial was to assess the impact of socio-ecological systems and resilience (SESR)-based strategies on dengue vector control in schools and neighbouring household communities in Kampong Cham province.

### Hypothesis

The trial aimed to show that a package of dengue vector control activities in schools and households, including biophysical interventions—such as guppy fish, mosquito gravid ovitraps, breeding site container covers and solid waste management—and community engagement activities—such as education and training, communication and behaviour change and participatory mapping—will significantly reduce entomological indicators for dengue in households in intervention areas when compared to households in control areas.

A publication by Echaubard et al. (2020) presents preliminary qualitative results from this trial pertaining to the implementation of the interventions and the building of adaptive capacity for dengue control in the community. [22] A forthcoming publication will present an evaluation of the community engagement activities of the trial. This publication presents an analysis of quantitative entomological results from the trial.

## Methods

### Ethics statement

Ethical approval was granted for the interventions and data collection by the National Ethics Committee for Health Research at the Ministry of Health in the Kingdom of Cambodia on 9th July 2018 (reference number 160) and was reapproved on 1st July 2019 (reference number 162).

The trial was conducted over 24 months from May 2018 to April 2020 in Kampong Cham, a province of 895,763 inhabitants in the central lowlands of Cambodia [23]. Kampong Cham was chosen because it has a dengue incidence rate of 1.6 cases per 1,000 people, one of the highest in Cambodia, and because it has similar environmental characteristics to other provinces with high dengue burdens [24]. Kampong Cham has two seasons: a rainy season running from May to November, when dengue incidence is higher, and a dry season for the remainder of the year [11].

Twenty village clusters were randomly selected in the Kampong Siam and Prey Chhor districts of Kampong Cham province, with an average of 161 households and 728 individuals per cluster. Clusters had to be at least 200 metres from the nearest household outside the cluster to avoid spillover effects as *Aedes aegypti* have an average dispersal range of 50 to 100 metres [25]. Every house within the cluster boundaries was invited to participate in the trial. Clusters were randomised into one of the two study arms on the basis of a “lucky draw”, with a representative from each village drawing a folded piece of paper with an arm number on it from a transparent bowl. Both trial arms received the standard vector control activities from the Ministry of Health, which consisted of thermal fogging with deltamethrin. The Intervention Arm additionally received entomological interventions and community engagement activities, as described below and discussed at length in Echaubard et al. (2020) [22]. [Table 1]. Note that a third study arm, also containing 10 randomly selected village clusters, was initially planned to receive only the entomological interventions, without the community engagement activities, but was subsequently abandoned due to incorrect application of interventions in that arm upon project commencement.

**Table 1:** Trial design: Interventions implemented in each trial arm.

Intervention type	Intervention Component	Intervention Description	Intervention Arm		Control Arm	
			8 Schools	10 Villages	8 Schools	10 Villages
**Biophysical**	**Biological larval control**	Guppy fish rearing, distribution, and placement in key household water-containers	✓	✓		
	**Adult *Aedes* control**	Distribution of mosquito Gravid Ovitraps for use in schools and households	✓	✓		
	**Breeding site container covers**	For water storage jars not suitable for guppy fish, lids of wood, metal or netting to prevent egg-laying	✓	✓		
	**Solid waste management**	Larval source control through improved solid waste management (empty tins etc)	✓	✓		
**Community engagement**	**Education and training**	Place-based educational campaign on dengue disease, vector biology, ecology and control, role of solid waste, clean water and health relationships	✓	✓		
	**Communication & Behaviour Change**	Communication for Behavioural Impact using multipronged communication channels including interpersonal communication through volunteers, folk or local media and mass media.	✓	✓		
	**Participatory mapping**	Map co-creation as a tool for community ownership of dengue decentralized surveillance and management	✓	✓		

### Biophysical interventions

#### (i) Provision of guppy fish

**Fig 1:**
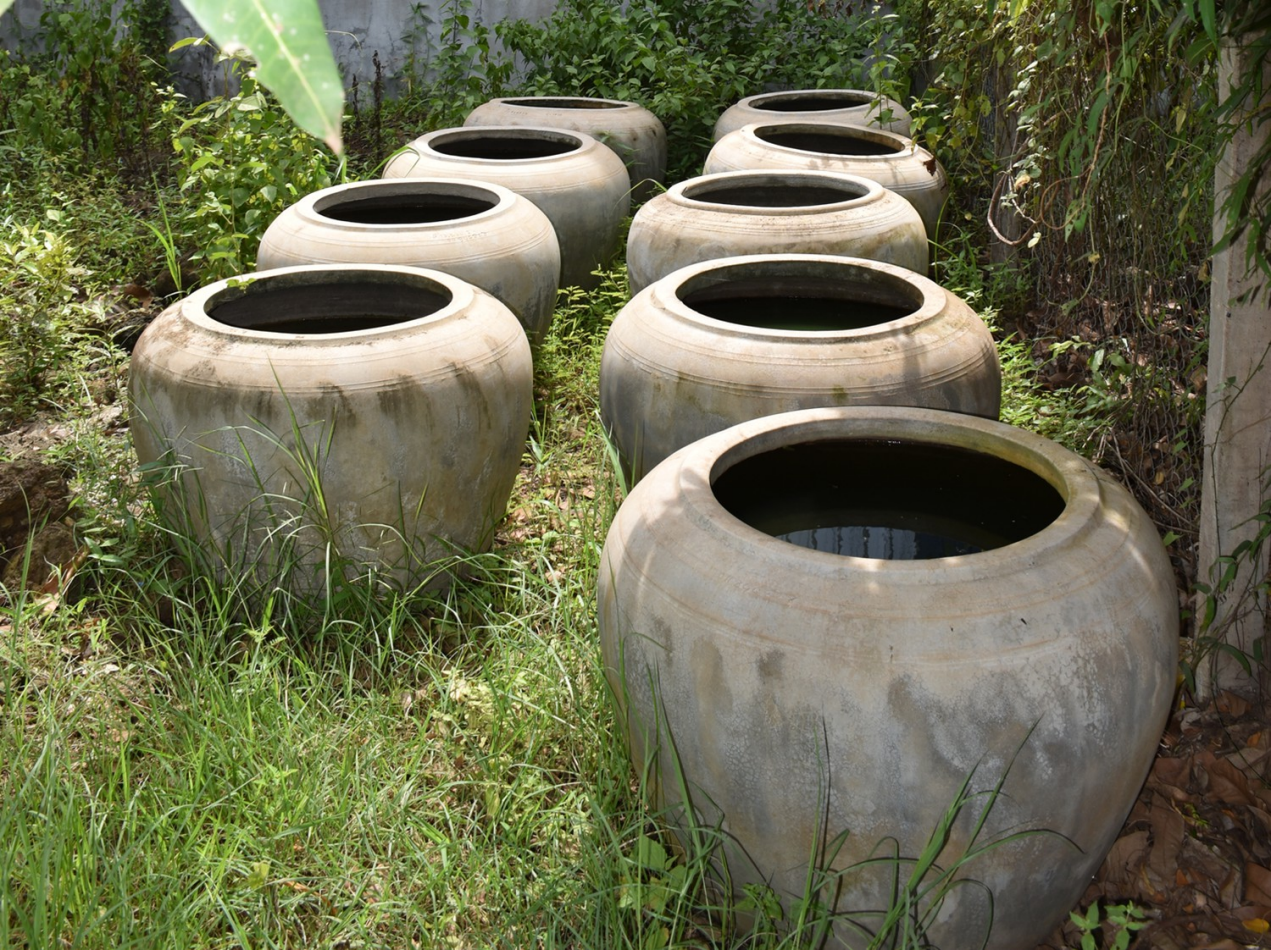
Example of a guppy fish farm established in large concrete water jars in a community health centre.

*Poecilia reticulata* are ornamental fish which are regarded as “bringers-of-good-luck” in Cambodian society and therefore readily accepted within households. Guppy fish “farms” were established at schools and community health centres (CHCs) in the 10 villages comprising the Intervention Arm. Three 400-litre water jars were provided to each of 8 schools; twenty 400-litre jars were provided to each of three CHCs; and three 400-litre jars were provided elsewhere in each of the 10 villages, generally at the households of Village Chiefs. The jars were filled with water and stocked with guppy fish that are fed with rice husks twice a day. [Fig 1]. Fish from these farms were supplied free of charge to school children from villages within the Intervention Arm to take home and place in outdoor water storage jars and tanks. The fish bred within these jars and tanks and the mature fry were used to stock and replenish other household water containers as required. A total of 13,200 fish were distributed to households in this way. Training regarding the value of the guppy fish and the methods involved in their rearing and maintenance was provided to 50 school teachers (who in turn provided training to their students), 47 community health workers (CHWs) and at 3 CHCs.

#### (ii) *Aedes* trapping

4,764 gravid ovitraps [26] of two designs, medium and small, as described in Echaubard et al. (2020) [22], were distributed to households and schools in the Intervention Arm. The medium traps were produced by CHWs in each village, while a Women’s Group was established and paid to manufacture the small traps. The lower halves of these traps were filled with water infused with dry grass for seven days to serve as attractant for gravid *Aedes* seeking oviposition sites [27]. Mosquitoes entering the trap were prevented from accessing the water by a layer of mosquito-netting midway up the trap. Mosquitoes inside would be trapped on sticky paper lining the wall of the upper chamber, with the paper being replaced at monthly intervals. Three traps (one medium size and two small size) were provided to each of the 1,656 households in the Intervention Arm and two medium-sized traps per room were provided in 80 rooms at the 8 schools in the Intervention Arm.

#### (iii) Solid waste management

Villagers in the Intervention Arm were shown examples during the Communication for Behavioural Impact (COMBI) activities described below of the empty discarded bottles, tins, old car tyres, coconut husks and similar objects which lie around many household yards and collect rainwater in which *Aedes* breed. As no municipal waste collection services exist, villagers were requested to collect such discarded materials and either burn or bury them. Schools were encouraged to hold regular clean-up sessions during which students collect such containers lying around the schoolyard and dispose of them. Reminders and checks were performed in person by CHWs at least once per semester.

#### (iv) Covering of open water containers

Villagers in the Intervention Arm were encouraged to cover any empty pots, jars or other containers in which rainwater could collect and to cover water-storage containers in which they did not want to place guppy fish, using either netting material, metal or wooden lids. Reminders and checks were performed in person by CHWs at least once per semester.

### Community engagement interventions

Social engagement, the promotion of community participation and the training of key behaviour change agents formed a large component of the study. The *a priori* hypothesis was that without community commitment and active community contribution to dengue vector control, the vector control interventions would fail. An integrated portfolio of activities was therefore implemented, as described below, with a particular focus on the engagement of school children as evidence from Kampong Cham province shows that schools represent a risk for vector-borne disease transmission [28].

#### (i) Education and training

Following consultation with a range of stakeholders, an “Instruction Lesson on Guppy fish as Dengue Vector Control Tool” was integrated into the National Health Education Curriculum of Ministry of Education, Youth and Sports. Training-of-trainers was provided to 50 school teachers and 110 “School Training Program for Teachers” manuals in the Khmer language were prepared and distributed. This training was based on a training needs assessment developed following in-depth discussions with school directors, teachers and students. 1,000 informational leaflets and 50 posters were prepared and distributed to school teachers and school libraries. A total of 1,700 school students participated in 300 health education sessions.

#### (ii) Communication for Behavioural Impact (COMBI)

In collaboration with the Ministry of Education, school curriculum departments and local authorities, 40 community-based health education sessions were organised involving 1,220 villagers, more than 60 percent of whom were women. Approximately 4,000 informal talks on dengue were delivered. Capacity building sessions for 47 community health workers (CHWs) at CHCs were held regularly to reinforce messaging and provide information materials.

#### (iii) Participatory mapping

325 villagers, mostly women, participated in 10 Participatory Epidemiological Mapping Meetings. These meetings involved the co-creation of community maps representing local perceptions of breeding site locations, zones of contact with mosquitoes, frequency, extent and timing of the movement of people, significant infrastructure enabling the presence of mosquitoes and general epidemiological data. The created maps were then used to focus subsequent vector control efforts, including the solid waste management activities described above [22]. Further details regarding community engagement interventions and measures of output will be provided in a future publication.

### Data collection

Intensive entomological surveys were conducted over four two-week periods in August 2018 (baseline), February 2019, August 2019 and February 2020. Four teams each comprising four people trained in *Aedes* sampling and data collection, supervised by a management team of two people, conducted the surveys. One person per team served as data-recorder, either on handwritten forms (one survey) or using ODK Collect (Get ODK Inc.) on cellphones (three surveys) [26].

A sample of 20 households per village cluster were surveyed in August 2018, August 2019 and February 2020, giving a total sample size of 400 households per survey, with 200 households per study arm. The sample was chosen by dividing the number of households in each cluster by 20 then surveying a house every multiple of the answer. For example, in a cluster of 200 households, every tenth household was sampled. Despite plans to also sample 400 households in February 2019, only 212 houses could be sampled due to staffing constraints. The number of houses sampled was the same in the Intervention and Control arms, however.

In each survey, all water containers in the selected houses were identified and categorised into one of 10 container types (drum, water storage jar, concrete tank, cement basin, small pot, flower vase/pot/try, tyre, can/bottle, miscellaneous-domestic use, other). Four of the container categories are typically used for domestic water storage—water storage jars, cement basins, cement tanks and drums—and the remainder are containers found in the area around houses which incidentally fill with water when it rains [29]. Each container was assessed for whether it contained mosquito larvae and/or pupae and whether it contained guppy fish. For containers smaller than 50 litres, the container was upturned and the water poured through a simple scoop net to collect all the larvae and pupae. For containers larger than 50 litres, upturning was impractical so samples of larvae and pupae were taken using a standardised five-sweep net method in which the net was swept five times anticlockwise beneath the surface of the water, and then one minute later swept once from the bottom of the container to the top [30]. Larvae and pupae were collected into labelled plastic bags containing water and sent to the laboratory for species identification and counting.

Three surveys of adult mosquitoes in households were undertaken, in August 2018, August 2019 and February 2020. No adult survey could be undertaken in February 2019 due to staffing issues. Adult mosquitoes were collected using portable aspirators, designed and manufactured in Phnom Penh by Mr Tho Setha, while they rested on walls. The aspirators were used for 10 minutes per house, starting in the bedroom and aspirating up and down the wall, from the floor to 1.5 metres in height, then continuing clockwise around the home. Collected adult mosquitoes were kept in the sealed and labelled cups as collected by the aspirators.

All samples were stored in refrigerators until microscopically identified, usually within 48 hours after collection. Each adult, larva and pupa was microscopically identified by a team of two trained and experienced entomologists from the Cambodian National Center for Parasitology, Entomology and Malaria Control. A 10% sample of collected specimens was stored in 70% alcohol before being further checked by entomologists at the United States Naval Medical Research Unit Two (NAMRU-2) in Phnom Penh for quality assurance, who confirmed the accuracy of the identifications.

### Data analysis

Data collected in August 2019 using handwritten forms was double data entered into ODK Collect. ODK Collect data from all four surveys was sent to ona.io (Ona Systems) for storage and later exported from ona.io to Microsoft Excel (Microsoft Corporation). Statistical analysis was performed in Stata 16 (StataCorp). Five main entomological indices were calculated:

1. Container Index: percentage of water containers infested with at least one mosquito larva and/or pupa of any species
2. Household Index: percentage of houses with at least one container infested with any species of mosquito larvae and/or pupae
3. Breteau Index: number of containers infested with at least one mosquito larva and/or pupa of any species per 100 houses inspected
4. Pupal Index: mean number of *Aedes aegypti* and *Aedes albopictus* pupae per 100 houses
5. Adult Index: mean number of adult female *Aedes aegypti* and *Aedes albopictus* per house

### Also calculated were

- Container Index by container type
- The proportion of eligible water containers containing guppy fish, by container type and study arm
- The proportion of eligible water containers infested with mosquito larvae and/or pupae, by container type and whether the container contained guppy fish

Because the five-sweep net method cannot collect all the pupae in containers larger than 50 litres, for calculation of the Pupal Index the total number of pupae in these containers was estimated using a multiplication factor based on container size, based on the work of Knox et al. (2007) [30].

### Statistical methods

Values of the Container and House indices for different study arms, being proportions, were compared statistically with a generalised linear model for binomial data with a log-link function, using a sandwich estimator to account for the effect of the clustering. The model tested the null hypothesis of no difference between the study arms. The same model was used to calculate confidence intervals for the indices. Values of the Breteau Index, Pupal Index and Adult Index for different study arms, being counts, were compared statistically with negative binomial regression which accounts for the effect of the clustering. The same model was used to calculate confidence intervals for the indices.

## Results

Results of the Container Index, House Index and Breteau Index are shown in Table 2. In the August 2018 and February 2019 surveys, there was no evidence of a difference in the three indices between the Control and Intervention Arms, with the exception of a significantly lower Breteau Index in the Control Arm than the Intervention Arm in August 2018 (p = 0.002). In the August 2019 and February 2020 surveys, there was very strong evidence that all three indices were lower in the Intervention Arm than in the Control Arm (all p-values = 0.001 or lower).

**Table 2:** Container Index, House Index and Breteau Index by survey time point.

	Containers	Infested containers	Houses	Infested houses	Container Index (95% CI)	p-value*	House Index (95% CI)	p-value*	Breteau Index (95% CI)	p-value**
**August 2018**										
Overall	2184	457	397	214	21 (19–23)	0.20	54 (47–62)	0.27	115 (101–131)	0.002
Intervention Arm	1386	276	199	115	20 (18–22)		58 (48–69)		139 (116–165)	
Control Arm	798	181	198	99	23 (19–27)		50 (41–60)		91 (75–111)	
**February 2019**										
Overall	866	142	212	83	16 (13–20)	0.91	39 (34–45)	0.21	67 (53–84)	0.33
Intervention Arm	476	79	106	45	17 (13–22)		42 (35–51)		75 (55–102)	
Control Arm	390	63	106	38	16 (11–23)		36 (30–43)		59 (43–83)	
**August 2019**										
Overall	1643	359	399	207	22 (16–29)	0.001	52 (43–63)	<0.001	90 (79–102)	<0.001
Intervention Arm	790	104	200	66	13 (9–19)		33 (26–42)		52 (42–64)	
Control Arm	853	255	199	141	30 (22–41)		71 (63–79)		128 (110–150)	
**February 2020**										
Overall	1275	262	400	174	21 (14–30)	<0.001	44 (30–63)	<0.001	66 (57–75)	<0.001
Intervention Arm	602	25	200	23	4 (2–7)		12 (7–19)		13 (8–18)	
Control Arm	673	237	200	151	35 (30–42)		76 (65–88)		119 (104–135)	

95% CI = 95% confidence interval.

*p-values for null hypothesis of no difference in Container Index or House Index between the two study arms, derived using a generalised linear model for binomial data with a log-link function and a sandwich estimator.

**p-value for null hypothesis of no difference in Breteau Index between the two study arms, derived using negative binomial regression.

**Fig 2:**
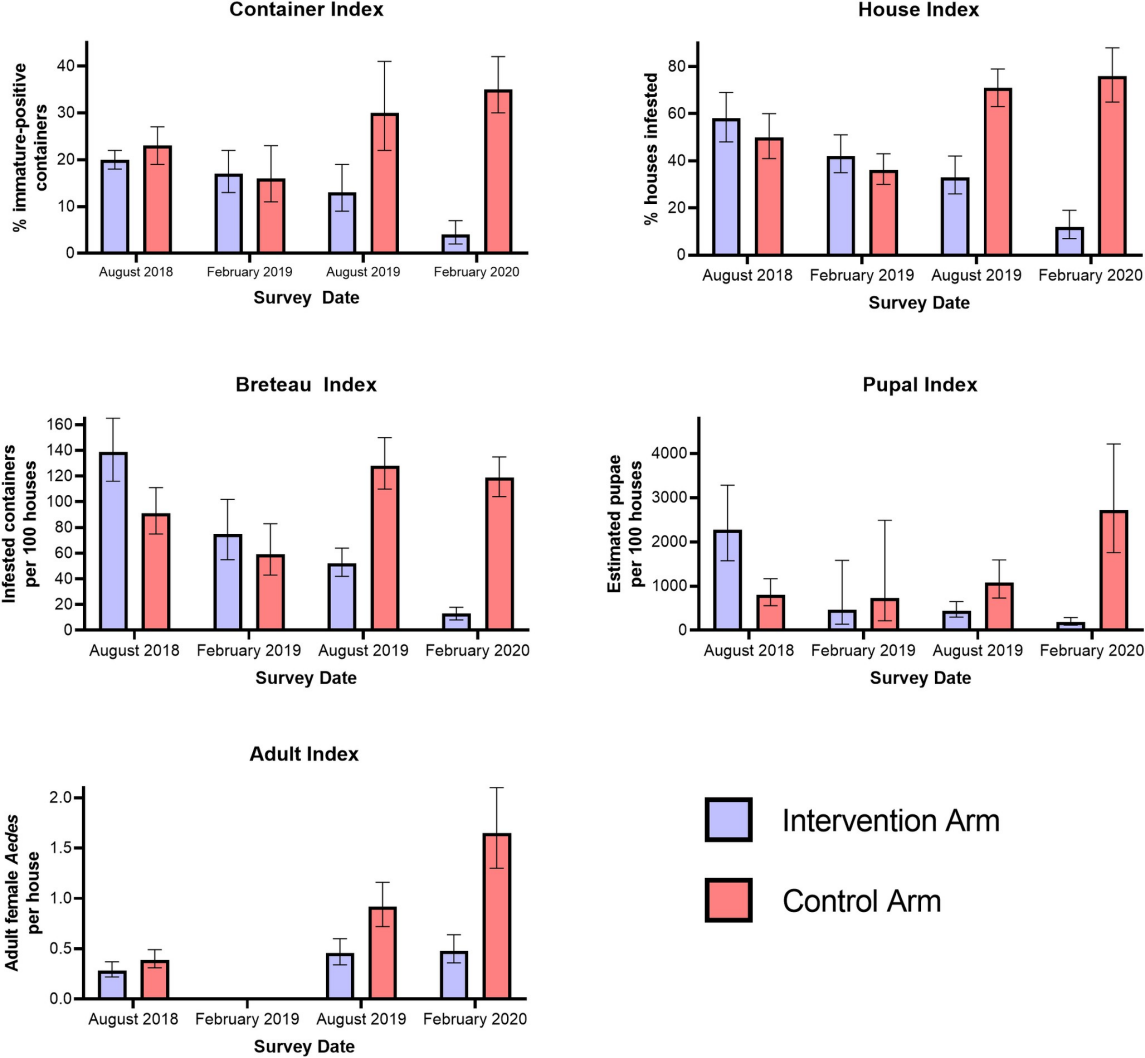
Container, House, Breteau, Pupal and Adult Indices for the Control and Intervention Arms by survey time point.

Results of the Pupal Index and Adult Index are shown in Table 3. There was very strong evidence that the Pupal Index was lower in the Control Arm than the Intervention Arm in the August 2018 survey (p<0.001) and no evidence of a difference between the arms in February 2019 (p = 0.07). There was no evidence of a difference in Adult Index between the arms in August 2018 (p = 0.60) and no data were available in February 2019. In the August 2019 and February 2020 surveys, there was very strong evidence that both indices were lower in the Intervention Arm than in the Control Arm (all p-values = 0.002 or lower). Results from all entomological indices are summarised in Fig 2.

**Table 3:** Pupal Index and Adult Index by survey time point.

	Pupae	Adults	Houses	Pupal index (95% CI)	p-value*	Adult Index** (95% CI_	p-value*
**August 2018**							
Overall	6154	135	400	1539 (1177–2010)	<0.001	0.34 (0.30–0.39)	0.07
Intervention Arm	4545	57	200	2272 (1572–3284)		0.28 (0.22–0.37)	
Control Arm	1610	78	200	805 (556–1165)		0.39 (0.31–0.49)	
**February 2019**							
Overall	1273		212	600 (253–1427)	0.60		
Intervention Arm	494		106	466 (137–1580)			
Control Arm	779		106	735 (217–2490)			
**August 2019**							
Overall	3022	274	399	757 (571–1004)	0.002	0.69 (0.58–0.81)	<0.001
Intervention Arm	878	91	200	439 (296–650)		0.46 (0.34–0.60)	
Control Arm	2144	183	199	1077 (729–1592)		0.92 (0.72–1.16)	
**February 2020**							
Overall	5816	426	400	1454 (1021–2070)	<0.001	1.07 (0.88–1.30)	<0.001
Intervention Arm	370	96	200	185 (118–290)		0.48 (0.36–0.64)	
Control Arm	5445	330	200	2723 (1758–4216)		1.65 (1.30–2.10)	

95% CI = 95% confidence interval.

*p-value for null hypothesis of no difference in Pupal Index or Adult Index between the two study arms, derived using negative binomial regression.

**data on Adult Index were unavailable for February 2019 survey

### Container surveys and presence of guppy fish

5,968 water containers were identified across the four surveys. The average number of containers per house was 5.64 in August 2018, 4.08 in February 2019, 4.10 in August 2019 and 3.18 in February 2020. The Container Index for the different container types is shown in Table 4.

**Table 4:** Container Index by container type, study arm and survey time point.

	Number of Positive Containers/Number of Containers Surveyed (Container Index)		
Type of Container	Total	Intervention Arm	Control Arm
**August 2018**			
Drum	14/62 (22.5)	5/34 (14.7)	9/28 (32.1)
Water Storage Jar	162/853 (19.0)	90/503 (17.9)	72/350 (20.6)
Concrete Tank	22/202 (10.9)	16/138 (11.6)	6/64 (9.4)
Cement Basin	74/295 (25.1)	46/189 (24.3)	28/106 (26.4)
Small Pot	12/35 (34.3)	7/21 (33.3)	5/14 (35.7)
Flower Vase/Pot/Tray	0/1 (0.0)	0/1 (0.0)	0/0 (0.0)
Tyre	16/39 (41.0)	10/27 (37.0)	6/12 (50.0)
Can/Bottle	2/3 (66.7)	2/3 (66.7)	0/0 (0.0)
Miscellaneous-Domestic Use	57/388 (14.7)	35/262 (13.4)	22/126 (17.5)
Other	98/306 (32.0)	65/208 (31.3)	33/98 (33.7)
Total	457/2184 (20.9)	276/1386 (19.9)	181/798 (22.7)
**February 2019**			
Drum	7/39 (17.9)	6/23 (26.1)	1/16 (6.3)
Water Storage Jar	67/376 (17.8)	40/196 (20.4)	27/180 (15.0)
Concrete Tank	8/95 (8.4)	5/64 (7.8)	3/31 (9.7)
Cement Basin	35 (24.0)	16/71 (22.5)	19/75 (25.3)
Small Pot	0/0 (0.0)	0/0 (0.0)	0/0 (0.0)
Flower Vase/Pot/Tray	3/3 (100.0)	1/1 (100.0)	2/2 (100.0)
Tyre	1/4 (25.0)	1/3 (33.3)	0/1 (0.0)
Can/Bottle	0/0 (0.0)	0/0 (0.0)	0/0 (0.0)
Miscellaneous-Domestic Use	10/122 (8.2)	5/76 (6.6)	5/46 (10.9)
Other	11/81 (13.6)	5/42 (11.9)	6/39 (15.4)
Total	142/866 (16.4)	79/476 (16.6)	63/390 (16.2)
**August 2019**			
Drum	6/47 (12.8)	0/11 (0.0)	6/36 (16.7)
Water Storage Jar	119/665 (17.9)	44/338 (13.0)	75/327 (22.9)
Concrete Tank	16/254 (6.3)	3/149 (2.0)	13/105 (12.4)
Cement Basin	65/298 (21.8)	20/155 (12.9)	45/143 (31.5)
Small Pot	0/1 (0.0)	0/1 (0.0)	0/0 (0.0)
Flower Vase/Pot/Tray	0/0 (0.0)	0/0 (0.0)	0/0 (0.0)
Tyre	27/45 (60.0)	5/11 (45.5)	22/34 (64.7)
Can/Bottle	4/5 (80.0)	0/0 (0.0)	4/5 (80.0)
Miscellaneous-Domestic Use	47/142 (33.1)	17/63 (27.0)	30/79 (38.0)
Other	75/186 (40.3)	15/62 (24.2)	60/124 (48.4)
Total	359/1643 (21.9)	104/790 (13.2)	255/853 (29.9)
**February 2020**			
Drum	7/34 (20.6)	0/11 (0.0)	7/23 (30.4)
Water Storage Jar	135/636 (21.2)	17/272 (6.3)	118/364 (32.4)
Concrete Tank	17/206 (8.3)	3/133 (2.3)	14/73 (19.2)
Cement Basin	80/316 (25.3)	4/142 (2.8)	76/174 (43.7)
Small Pot	0/0 (0.0)	0/0 (0.0)	0/0 (0.0)
Flower Vase/Pot/Tray	2/4 (50.0)	0/0 (0.0)	2/4 (50.0)
Tyre	1/1 (100.0)	1/1 (100.0)	0/0 (0.0)
Can/Bottle	0/0 (0.0)	0/0 (0.0)	0/0 (0.0)
Miscellaneous-Domestic Use	7/42 (16.7)	1/28 (3.6)	6/14 (42.9)
Other	13/36 (36.1)	0/16 (0.0)	13/20 (65.0)
Total	262/1275 (20.5)	25/602 (4.2)	237/673 (35.2)

Of the four main types of container eligible for guppy fish (drum, drinking water storage jar, concrete tank and cement basin), 669 (27.6%) of the 2,426 containers in the Intervention Arm contained the fish across the four surveys. 136 (6.5%) of the 2,095 containers in the Control Arm contained the fish. The proportion of containers containing guppy fish increased from 10.1% in August 2018 to 40.7% in February 2020 in the Intervention Arm and from 8.6% in August 2018 to 9.3% in February 2020 in the Control Arm. [Table 5].

**Table 5:** Proportion of eligible water containers containing guppy fish, by container type, study arm and survey time point.

	Number of containers with guppy fish/Total number of containers (%)	
Type of Container	Intervention Arm	Control Arm
**August 2018**		
Drum	3/34 (8.8)	0/28 (0.0)
Water Storage Jar	63/503 (12.5)	40/350 (11.4)
Concrete Tank	16/138 (11.6)	3/64 (4.7)
Cement Basin	6/189 (3.2)	4/106 (3.8)
Total	88/864 (10.1)	47/548 (8.6)
**February 2019**		
Drum	1/23 (4.4)	0/16 (0.0)
Water Storage Jar	59/196 (30.1)	16/180 (8.9)
Concrete Tank	10/64 (15.6)	0/31 (0.0)
Cement Basin	5/71 (7.0)	2/75 (2.7)
Total	75/354 (21.2)	18/302 (6.0)
**August 2019**		
Drum	4/11 (36.4)	0/36 (0.0)
Water Storage Jar	141/338 (41.7)	5/327 (1.5)
Concrete Tank	64/149 (43.0)	2/105 (1.9)
Cement Basin	70/155 (45.2)	5/143 (3.5)
Total	279/650 (42.9)	12/611 (2.0)
**February 2020**		
Drum	8/11 (72.7)	1/23 (4.3)
Water Storage Jar	132/272 (48.5)	32/364 (8.7)
Concrete Tank	37/133 (27.8)	4/73 (5.5)
Cement Basin	50/142 (35.2)	22/175 (12.6)
Total	227/558 (40.7)	59/634 (9.3)
Overall Total	669/2426 (27.6)	136/2095 (6.5)

Of the 805 containers of the four main types eligible for guppy fish which contained guppy fish across the four surveys, 32 (4.0%) were infested with larvae and/or pupae. Of the 3,719 containers identified across the four surveys which did not contain guppy fish, 802 (21.6%) were infested with larvae and/or pupae. [Table 6].

**Table 6:** Number and proportion of containers infested with mosquito larvae and/or pupae, by container type, whether or not the container contains guppy fish and survey time point.

	Number of positive containers/Total number of containers (%)	
Type of Container	Containers with guppy fish	Containers without guppy fish
**August 2018**		
Drum	0/3 (0.0)	14/59 (23.7)
Water Storage Jar	1/103 (1.0)	161/750 (21.5)
Concrete Tank	0/19 (0.0)	22/183 (12.0)
Cement Basin	0/10 (0.0)	74/285 (26.0)
Total	1/135 (0.7)	271/1277 (21.2)
**February 2019**		
Drum	0/1 (0.0)	7/38 (18.4)
Water Storage Jar	3/75 (4.0)	64/301 (21.2)
Concrete Tank	1/10 (10.0)	7/85 (8.2)
Cement Basin	1/7 (14.3)	34/139 (24.5)
Total	5/93 (5.4)	112/563 (19.9)
**August 2019**		
Drum	0/4 (0.0)	6/43 (14.0)
Water Storage Jar	0/146 (0.0)	119/519 (22.9)
Concrete Tank	0/66 (0.0)	16/188 (8.5)
Cement Basin	0/75 (0.0)	65/223 (29.2)
Total	0/291 (0.0)	206/973 (21.2)
**February 2020**		
Drum	0/9 (0.0)	7/25 (28.0)
Water Storage Jar	11/164 (6.7)	124/472 (26.3)
Concrete Tank	2/41 (4.9)	15/165 (9.1)
Cement Basin	13/72 (18.1)	67/244 (27.5)
Total	26/286 (9.1)	213/906 (23.5)
Overall Total	32/805 (4.0)	802/3719 (21.6)

## Discussion

This large-scale cluster-randomised trial provides very strong evidence that a package of biophysical and community engagement interventions was effective in reducing entomological indicators for dengue vectors compared to the control group in Kampong Cham province, Cambodia. However, the extent to which reductions in these entomological indicators will result in reductions in the number of dengue cases cannot be inferred from these results.

Results from August 2018 and February 2019, the first two of four household surveys, showed no significant difference between the study arms for the five entomological indices, with the exception of the findings of significantly lower Breteau and Pupal Indices in the Control Arm than in the Intervention Arm in August 2018. These findings appear to be an artefact of the way the two indices are calculated: the Container Indices were similar for the two arms but substantially fewer containers were identified in the Control Arm (798 in 198 houses = 4.0 per house) than in the Intervention Arm (1386 in 199 houses = 7.0 per house), meaning that the number of infested containers per 100 houses was inevitably lower in the Control Arm, as was the mean number of pupae per 100 houses. In the three subsequent surveys, the number of containers identified in each arm was more evenly distributed between the Control and Intervention Arms (3.7 and 4.5 per house in February 2019, 4.3 and 4.0 per house in August 2019 and 3.4 and 3.0 per house in February 2020). This could suggest that the high number of containers per house in the Intervention Arm in August 2018 was due to sampling variation rather than representing an underlying difference in the number of containers per house between the arms. However, it is also possible that the solid waste management part of the biophysical interventions was responsible for reducing the number of containers per house in the Intervention Arm over the course of the study.

It is unsurprising that results from August 2018 and February 2019 showed no significant difference between the arms because operational delays meant that implementation of many of the interventions, including distribution of guppy fish and traps, as well as training and messaging, did not begin until January 2019. Results from August 2019 and February 2020, after the interventions had been up and running for some time, showed very strong evidence of a reduction in all five entomological indices in the Intervention Arm when compared with the Control Arm (all p-values 0.002 or lower). A study by the Asian Development Bank of the use of guppy fish and community engagement for dengue vector control in Cambodia found a similar lag period: three months after the start of the project, there was a difference in Container Index between intervention and control groups but the strongly significant difference between the two groups did not appear until a survey six months into the project [21].

It is notable that the entomological indices remained relatively low in the Intervention Arm in the August 2019 and February 2020 surveys, even as they increased substantially in the Control Arm in those surveys. The increases in the Control Arm may reflect that 2019 was the year with the largest number of global dengue cases on record [31], with dengue outbreaks across the Asia-Pacific region [32] and substantially more dengue cases reported in Cambodia than the 5-year average [33]. However, the direct link between regional dengue cases and entomological indices in this study is difficult to verify. Additionally, it is notable that the Adult Index in the Intervention Arm was higher in February 2020 than in August 2018, despite the significant difference between the Arms. This may suggest that the statistically significant reduction in immature mosquito indices were sufficient to prevent substantial rises in the adult mosquito population in the Intervention Arm but not to reduce the overall general increase in the adult mosquito population taking place at the time.

The use of the guppy fish *Poecilia reticulata* in household water containers was found to be highly acceptable to the households in the surveys, and guppies were present in almost 10% of containers in the August 2018 survey, before the project began to distribute the fish, showing a background use of guppy fish in Kampong Cham province which is likely related to previous studies of guppies in the same province and same villages [17,21]. Guppy fish appeared to be effective predators of immature mosquito stages, although they were unable to eat all the immature stages in their containers: across the four surveys, the Container Index in containers with guppies was 4.0% compared with 21.6% in containers without guppies, an 81% reduction.

In the February 2020 survey, guppies were found in 40.7% of eligible containers in the Intervention Arm. This compares with the earlier cluster-randomised trial in the same villages in which guppy coverage was approximately 60–70% [17] and with non-randomised studies in Cambodia in which *Poecilia reticulata* were found in 57% of eligible containers [20] and 88% of eligible containers [21], respectively. Guppy fish coverage may have been lower in this project than previous projects due to the passive nature of guppy fish distribution, which relied in particular on school children taking fish home to their households. However, comparing the Container Index between Intervention and Control Arms in these studies, guppy coverage does not appear to be directly correlated with the extent of the reduction in Container Index in the Intervention Arm. In the February 2020 survey in this study, the Container Index was 89% higher in the Control Arm than the Intervention Arm. In the other cluster-randomised trial, the Container Index was 38% higher in the Control Arm than the Intervention Arm [17]. In the non-randomised study with 57% guppy coverage, it was 77% higher in the Control than the Intervention Arm [20], while in the study with 88% guppy coverage, it was approximately 90% higher in the Control than the Intervention Arm [21]. As the packages of interventions used differed between the studies, it is difficult to identify the precise reasons for the variable results of the studies but all of the studies in Cambodia which utilised guppy fish saw significant reductions in the Intervention Arms compared with the Control Arms, suggesting that the fish are effective larval control agents.

As well as missing data for the Adult Index in February 2019, our study had some limitations. The use of multiplication factors to estimate the Pupal Index for containers which are too large to upturn is common practice [30] but introduces biases which are difficult to quantify. However, the study arms had similar proportions of the different container types in all four surveys and so were multiplied in proportion to each other, maintaining the relative differences between the arms.

A further limitation of our study is that our outcomes were entomological indicators for dengue, rather than cases of dengue. A 2004 review for the WHO [34] found that while the House, Container and Breteau indices are useful measures of the impact of interventions on mosquito immature stages, they are not reliable proxies for the abundance of adult vectors. Similarly, the relationship between the number of sampled adult mosquitoes and the overall number of adult mosquitoes per house is difficult to quantify, as is the relationship between the overall number of adult mosquitoes and the risk of dengue transmission in humans, [34,35]. However, measuring epidemiological outcomes was not possible in this study due to funding limitations.

In conclusion, a comprehensive package of biophysical and community engagement interventions was very effective in reducing entomological indicators for dengue in Kampong Cham province, Cambodia, although the extent to which these reductions resulted in practically significant reductions in the adult mosquito population cannot be inferred from these results. A previous cluster-randomised trial of similar interventions conducted in the same villages showed promising results [17]. Results from this study add further to the evidence that such a package of interventions is effective in reducing entomological indicators for dengue in this province of Cambodia. The package of interventions should be trialled in other locations and future studies should aim to collect both entomological and epidemiological data, to ascertain the relationship between efficacy in entomological impact and the extent of suppression of dengue cases, which cannot be inferred from our results [34,35]. Future studies should also aim to assess the relative contributions of the biophysical and community engagement domains of the intervention to refine our understanding of their impacts and finetune their delivery.
